# Molecular characterization and phylogenetic analysis of major envelope protein gene (B2L) and ATPase protein gene (A32L) of orf virus isolates from goats in Southern, Thailand

**DOI:** 10.1371/journal.pone.0340195

**Published:** 2026-01-30

**Authors:** Wongkae Watjiranon, Chya Vannakovida, Thanidtha Te-Chaniyom, Natakorn Nokchan, Syriam Sooksawasdi Na Ayudhya

**Affiliations:** 1 Veterinary Research and Development Center (Upper southern region), Nakhon Si Thammarat, Thailand; 2 Faculty of Veterinary Science, Prince of Songkla University, Songkhla, Thailand; 3 Translational Medicine Research Center, Faculty of Medicine, Prince of Songkla University, Songkhla, Thailand; 4 Department of Biomedical Sciences and Biomedical Engineering, Faculty of Medicine, Prince of Songkla University, Songkhla, Thailand; Tsinghua University, CHINA

## Abstract

Orf virus (ORFV), a member of the *Poxviridae* family, causes contagious ecthyma (CE), a viral skin disease in small ruminants. Due to its self-limiting nature, low mortality rate and economic consequences, CE is considered as a neglected disease, resulting in underreporting in Thailand. Despite its global presence, the genetic characterization of ORFV in Thailand, especially in the southern region where there is high density of goat farming, is largely unknown. To address the knowledge gap, we conducted genetic and evolutionary analysis including phylogenetic, BEAST, and discriminant analysis of principal components (DAPC), on ORFV isolated from Southern Thailand, utilizing conserved B2L gene (major envelope protein) and A32L gene (C-terminal ATPase protein). All suspected CE meat goats across 2 farms in Songkhla and Pattani provinces in 2024 tested ORFV positive via PCR using ORFV-specific primers. These isolates along with a positive control isolate (Pattani 2020) were used for molecular characterization and genetic analysis. The 2024 isolates clustered with Malaysian strains based on phylogenetic and population analysis. BEAST analysis of these isolates further indicated a shared evolutionary origin around 2007, suggesting transboundary spread. Although the 2024 isolates were highly related, some differentiation observed for both genes and they evolved separately from 2020 Pattani isolate suggesting ongoing local evolution and genetic variation in the regional population. Moreover, the Pattani 2020 isolate was phylogenetically distinct and revealed a distinct ancestral origin, suggesting a separate introduction event into the region. Notably, heterogeneity in C-terminal region of ATPase gene was observed, including 4 amino acid deletions in Songkhla 2024 and Pattani 2020 isolates, and unique substitutions (G258S and G260S) in Pattani 2024 which may cause a distinct cluster based on DAPC analysis. Collectively, our findings indicate diverse ORFV evolution in Southern Thailand, necessitating continued molecular surveillance and genetic characterization to improve national control strategies.

## Introduction

Contagious ecthyma (CE) is a viral skin disease in small ruminants caused by the orf virus (ORFV). Infected animals develop crusty scabs, typically located at the mucocutaneous junctions of the lips, nose, and mouth. The lesions begin as macules and papules, then progress to nodular or papillomatous growths, eventually forming thick brown scabs. The disease spreads through direct contact with infected animals or contaminated environments. Lesions are usually self-limiting and resolve within 3–6 weeks [1,2]. ORFV is also a zoonotic virus, meaning it can be transmitted to humans primarily through direct contact with infected sheep or goats. Individuals at higher risk include veterinarians, farmers, and animal handlers [3,4]. In most human cases, the infection remains localized and resolves without treatment. However, the condition in immunocompromised individuals may be more severe or prolonged [5].

Orf virus is a large, double-stranded DNA virus (134–139 kbp) belonging to the genus Parapoxvirus within *Poxviridae* family [1]. Similar to other poxviruses, the ORFV genome consists of a highly conserved central core region and variable terminal regions [1]. The central region of the viral genome contains essential genes involved in replication, transcription, and viral assembly [6]. In contrast, genes associated with virulence, tissue tropism, and virus-host interactions are typically located in the variable terminal regions [7].

Natural outbreaks of suspected ORFV infections are commonly observed in goats. Although the mortality rate of CE is generally low, the disease has a high morbidity rate. Infections in young animals (under 2 months old), those with secondary bacterial infections, or immunocompromised individuals can increase mortality rates by 20–50% [2,8–10]. Due to its self-limiting nature and endemic nature of the disease in Thailand, ORFV is often underdiagnosed. However, accurate diagnosis is essential, as other viral diseases in goats such as foot-and-mouth disease (FMD), sheep pox, goat pox, peste des petits ruminants, and bluetongue can produce similar oral lesions and clinical signs [11]. Therefore, specific laboratory diagnosis for ORFV is necessary, especially in endemic areas.

Multiple strains of ORFV have been circulating in small ruminants worldwide [12–20] and have exhibited genetic variation including recombination [21]. Contagious ecthyma disease in ruminants was first reported in Thailand in 1981, based on clinical and pathological diagnoses [22]. However, surveillance of CE in goats is currently limited, largely due to the disease’s self-limiting nature, low mortality with low economic loss. In addition, the ORFV detection relies mostly on clinical diagnosis leading to underreporting of the disease [23], and thereby hindering the knowledge on the virus’s epidemiology and genotyping over many years. A specific case of CE in goats was reported in Pattani in 2014 by pathological with molecular confirmation, but the specific virus genotyping was not performed limiting the epidemiological utility of the report [24]. Together, the overall genetic and molecular characterization of ORFV in Thailand remains largely unknown, particularly in the southern region where there is a high density of goat farming.

For molecular characterization and phylogenetic analysis, highly conserved regions of the ORFV genome are commonly targeted, including the B2L [19,25], F1L [25,26], and E3L [27,28] genes of which the B2L gene being the most frequently studied [29]. The B2L gene encodes a major envelop protein which is a key immunogenic protein p37K and a homologue to the vaccinia virus p37K envelope protein antigen [30]. Another important gene, A32L, is located near the right terminus of the ORFV genome [28] and exhibits genetic variation including substitutions and deletions [17]. The gene encodes a viral ATPase protein involved in viral DNA packaging [28,31] and contains 5 functional motifs which are conserved parts of the gene residing in N-terminal region [28,32,33]. Unlike the N-terminal region, the C-terminal region of the ATPase protein exhibits high heterogeneity, making it particularly useful for distinguishing between different ORFV strains [28].

Here, we present the first genetic sequences, phylogenetic analysis and molecular characterization of ORFV isolates from goats in Songkhla and Pattani provinces, Southern Thailand. Our findings provide new insights into the genotypes and molecular features of ORFV circulating in the region, contributing to improved surveillance and more effective control strategies.

## Materials and methods

### Epidemiological investigation and sample collection

Six suspected cases of CE presenting with lip skin lesions were investigated on 2 distinct goat farms located in Khlong Hoi Kong district, Songkhla Province, and Nong Chik district, Pattani Province in 2024 during an active surveillance by a veterinarian-instructor during a clinical study class of the Faculty of Veterinary Science, Prince of Songkla University from May to November 2024. On the Songkhla farm, 2 goats with CE symptoms were identified in the nursery unit (0–3 months old, n = 25). On the Pattani farm, 3 symptomatic goats were detected in the nursery unit (0–3 months old, n = 54) and 1 in the Saanen/adult unit (>3 months old, n = 53) (Table 1). The affected farms reported no history of new animal introductions within the preceding 6 months, covering an incubation period of CE. The owners also reported that they had observed scab lesions earlier. Due to its self-limiting nature’s disease, those affected animals did not receive any treatment. Separately, a CE case reported in Kok Poh district (Pattani) in 2020 was investigated by veterinarians from the diagnostic unit, Veterinary Research and Development Center (Upper southern region) following a direct report from a farmer, while the outbreak was not investigated further. The demographic data of affected farms and animals was reported in Table 1.

**Table 1:** Summary of orf virus-affected farms and animals.

Farm	Province	District	No. of affected animals	Isolate name	Age	Sex	Breed	House
1	Pattani	Kok Poh	1	Pattani/Thailand/goat/2020^a^	1 y	Female	Cross breed (Katjang and Boer)	Cross breed/adult house (n = 9)
2	Songkhla	Khlong Hoi Kong	1	Songkhla K5920/Thailand/goat/2024	2 w	Female	Anglo-nubian	Nursery house (n = 25)
			2	Songkhla K5921/Thailand/goat/2024				
3	Pattani	Nong Chik	1	Pattani 21/Thailand/goat/2024	1 m	Female	Saanen	Nursery house (n = 54)
			2	Pattani 23–65/Thailand/goat/2024	1 m	Female	Alpine	
			3	Pattani 83–65/Thailand/goat/2024	1m	Female	Saanen	
			4	Pattani 91298/Thailand/goat/2024	1 y	Female	Saanen	Saanen/adult house (n = 53)

The characteristics of orf virus-affected farms and animals, including the number of affected animals and management system. Affected animals are defined as those with scab lesions at the mouth but did not died. The number of affected animals is reported per farm. (No. = number, n = number, y = year, m = month).

^a^Positive control isolate.

**Fig 1:**
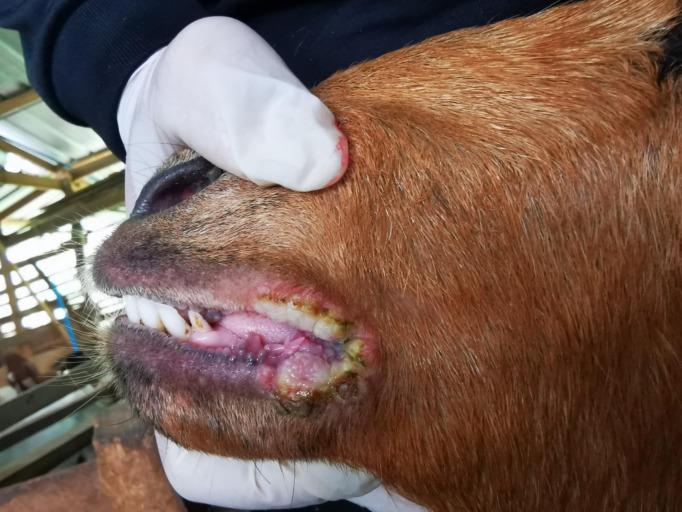
Skin lesions at the mouth of a goat suspected contagious ecthyma caused by orf virus infection. Scabby mouth lesions showing wheals, ecthyma, pustules, or scales at the lips of a goat.

Scab lesions were collected from clinically affected goats showing characteristic CE lesions, including wheals, pustules, ecthyma, and scaly crusts (Fig 1). Samples were obtained from the lips and nostrils using sterile scalpels and stored at –20 °C until further analysis.

Field works were granted permission from Small Ruminant Research and Development Center, Prince of Songkla University and Pattani Livestock Research and Breeding Center. A DNA sample (isolate Pattani/Thailand/goat/2020) and farm management data from a meat goat farm in Kok Poh district, Pattani, were provided by the Veterinary Research and Development Center (Upper Southern Region) (Table 1). This isolate served as a positive control in the study, and farmer permission was granted. Protocols were approved by the Institute Animal Care and Use Committee, Prince of Songkla University (project license number: MHESI 68014/637 and reference number: AG041/2024).

### Sample preparation

Tissue samples were homogenized with phosphate buffer saline (PBS), pH 7.0–7.4 supplemented with antibiotic and antifungal and adjusted to be 10% w/v suspension. The homogenized suspension was centrifuged at 3000 rpm for 10 minutes, collected the supernatant samples, and stored them at −20 °C until laboratory analysis.

### DNA extraction, screening polymerase chain reaction (PCR) and quantitative real-time PCR (qPCR)

Viral DNA was extracted from the supernatant using commercially available kit (DNeasy Blood & Tissue Kits, Qiagen, Germany) following the manufacturer’s instruction. The extracted DNA was eluted with a 50 µL elution buffer and stored at −20 °C. All samples were screened and confirmed by ORFV specific PCR [11] (Table 2), using Taq PCR Master Mix kit (Qiagen) and thermal cycler (Bio-gener, China). Pattani/Thailand/goat/2020 was used as an ORFV-PCR positive control in ORFV screening PCR. The PCR was performed in a 25 µL volumes, and the cycling reactions consist of initial denaturation at 94 °C for 5 min, followed by 35 cycles of 94 °C for 30 s, 55 °C for 30 s and 72 °C 45 s, and final extension 72 °C for 10 min. The PCR products were analyzed by electrophoresis on 2% agarose gel. This protocol allows to ruled out ORFV from FMDV, sheeppox virus, goatpox virus, bluetounge virus, and peste des petits ruminant virus. For each PCR run, instead of DNA template, water was used for a negative control.

**Table 2:** Primers for screening PCR, B2L and A32L gene amplification.

No.	Primer	Sequence 5’to 3’	Application	Product size	Ref.
1	ORFVF	CGAACTTCCACCTCAACCACTCC	Screening	507	[11]
2	ORFVR	CCTTGACGATGTCGCCCTTCT			
3	CaPV-074F1	AAAACGGTATATGGAATAGAG TTGGAA	Screening	–	[34]
4	CaPV-074R1	AAATGAAACCAATGGATGGGA TA			
5	CaPV-074P1	6FAMTGGCTCATAGATTTCCTMGBNFQ			
6	ORFV-B2Lf-For	GACCTTCCGCGCTTTAATTT	PCR sequencing	1210	[16]
7	ORFV-B2Lf- Rev	CCCGCCTGCTAAAAGACT			
8	ORFV-B2Li-For	GTCCGCGTTCTTCCACTC	PCR sequencing	554	[16]
9	ORFV-B2Li-Rev	GCGGGCGTCAACTACTACA			
10	ORFV-A32f-For	CTCCATTTAGAGGCCGTGAG	PCR sequencing	1098	[16]
11	ORFV-A32f-Rev	CGTGTTATGTGCCATCTTGC			
12	ORFV-A32i-For	GGTCGAGACAGCGCTTGA	PCR sequencing	509	[16]
13	ORFV-A32i-Rev	CGTCTACAACGCCGCCTAC			

All samples were ruled out from capripoxvirus using capripox-specific primers and probe (Table 2), using FastStart Essential DNA Probes Master (Roche) and real-time qPCRSoft cycler (Analytik jena, German) according to previous protocol [34]. Briefly, the quantitative real-time PCR (qPCR) was performed in a 20 µL volumes, contained 10 µL 2x master mix, 10 µM of each capripoxvirus primer and probe, 5 µL of template and water to 20 µL. The amplification program consists of pre-incubation at 95 °C for 10 min, followed by 45 cycles of 95 °C for 15 s and 60 °C for 45 s. The results were analyzed by absolute quantification of the threshold cycle. The cut-off CT value is at 37.

### Amplification and sequencing of B2L and A32L genes

Primers for DNA amplification specific for B2L and A32L genes were described by Gelaya et al. [16] (Table 2). The conventional PCR was performed using OmniPCR Supermix w fluorescent dye (Biohelix) and real-time system cycler (Bio-rad, USA). The PCR amplified condition for B2L gene was: initial denaturation at 95 °C for 5 min, followed by 35 cycles of 95 °C for 50 s, 52 °C for 60 s, and 72 °C for 90 s, and final extension at 72 °C for 7 min. The PCR amplification condition for A32L gene is similar with the former protocol, except annealing temperature is at 55 °C for 60 s. The PCR products were checked using electrophoresis on 1.5% agarose gel. The amplified products of B2L and A32L genes were purified using gel PCR extraction kit (Geneaid). Sequencing of the products was performed using the primer set number 6–13 (Table 2). The PCR products were sequenced using Abiseq PCR and sanger sequencing.

### Genotypic characterization, phylogenetic analysis and pairwise single nucleotide polymorphism distances

The sequences were assembled and edited using BioEdit software (V.7.2) [35]. The ORFV isolates in the GenBank that exhibit the highest similarity to Southern Thailand sequences were identified using the online BLAST program and were included in the phylogenetic analysis. The full-length B2L and A32L gene sequences of Southern Thailand isolates and other reference sequences retrieved from GenBank (Table 3) were aligned using Clustal W in BioEdit software (V.7.2). Phylogenetic trees of B2L and A32L genes were constructed by maximum-likelihood with optimal model selection and 1000 bootstrap replicates using MEGA12 software. All sequences obtained from this study were submitted to GenBank under accession number PV173479-PV173492.

**Table 3:** Representative Parapoxvirus B2L and A32L gene nucleotide sequences retrieved from GenBank using for genetic characterization and phylogenetic tree construction.

No.	Isolate name	Gene name	Country	Year	GenBank accession No.
1	FJ YT (KU199831)/China/goat/2014	B2L	China	2014	KU199831
2	NE2 (JN088051)/Brazil/goat/1993	B2L	Brazil	1993	JN088051
3	Assam (JN846834)/India/goat/2009	B2L	India	2009	JN846834
4	BPSV (AY424973)/USA/sheep	B2L	USA	–	AY424973
5	UPM-2/14 (KR024024)/Malaysia/goat/2014	B2L	Malaysia	2014	KR024024
6	UPM/HSN-20 (MW537048)/Malaysia/goat/2018	B2L	Malaysia	2018	MW537048
7	UPM-01-F1L (OP279270)/Malaysia/goat/2020	B2L	Malaysia	2020	OP279270
8	PCPV (AY424972)/USA/sheep	B2L	USA	–	AY424972
9	Yunnan/YNSLi (PP733997)/China/goat/2023	B2L	China	2023	PP733997
10	UPM-3/18 (OK169620)/Malaysia/goat/2018	B2L	Malaysia	2018	OK169620
11	UPM-3/14 (KR024025)/Malaysia/goat/2014	B2L	Malaysia	2014	KR024025
12	UPM-1/14 (KR024023)/Malaysia/goat/2014	B2L	Malaysia	2014	KR024023
13	D (JN088052)/Brazil/sheep/1992	B2L	Brazil	1992	JN088052
14	Korea (GQ328006)/Korea/goat/2009	B2L	Korea	2009	GQ328006
15	FJ-SJ2 (KC568397)/China/goat/2012	B2L	China	2012	KC568397
16	FJ-2402 (PP805860)/China/goat/2024	B2L	China	2024	PP805860
17	NZ2 (DQ184476)/New Zealand/sheep/1987	A32L	New Zealand	1987	DQ184476
18	GO (KP010354)/China/goat/2012	A32L	China	2012	KP010354
19	Nantou (EU327509)/Taiwan/goat/2006	A32L	Taiwan	2006	EU327509
20	Assam (JN183069)/India/goat/2010	A32L	India	2010	JN183069
21	Adet/O01(KT438531)/Ethiopia/sheep/2012	A32L	Ethiopia	2012	KT438531
22	SJ1 (KP010356)/China/goat/2012	A32L	China	2012	KP010356
23	Debre zeit/O01 (KT438543)/Ethiopia/sheep/2012	A32L	Ethiopia	2012	KT438543
24	Debre zeit/O02 (KT438544)/Ethiopia/sheep/2012	A32L	Ethiopia	2012	KT438544
25	ATARC/O01 (KT438540)/Ethiopia/sheep/2010	A32L	Ethiopia	2010	KT438540
26	ATARC/O01 (KT438539)/Ethiopia/sheep/2008	A32L	Ethiopia	2008	KT438539
27	Adet/O02 (KT438532)/Ethiopia/sheep/2012	A32L	Ethiopia	2012	KT438532
28	OV-IA82 (AY386263)/USA/sheep/1982	A32L	USA	1982	AY386263
29	SA00 (AY386264)/USA/goat/2000	A32L	USA	2000	AY386264
30	UPM/HSN-20 (MW537048)/Malaysia/goat/2018	A32L	Malaysia	2018	MW537048
31	India MP (MT332357)/India/goat/2017	A32L	India	2017	MT332357
32	Dhamapuri (MH766377)/India/goat/2018	A32L	India	2018	MH766377
33	Adet/O03 (KT438533)/Ethiopia/goat/2012	A32L	Ethiopia	2012	KT438533
32	PCPV (GQ329670)/New Zealand/bovine/1963	A32L	New Zealand	1963	GQ329670
33	BPSV (AY386265)/USA/bovine/2004	A32L	USA	2004	AY386265

Multiple sequence alignment (MSA) of the B2L and A32L genes was conducted using MEGA 7.0 [36], and pairwise single nucleotide polymorphism (SNP) distances were subsequently calculated with snp-dists v0.8.2 available from https://github.com/tseemann/snp-dits.

### Bayesian evolutionary analysis

For the evolutionary analysis of ORFV isolates circulating in Southern Thailand, we constructed a time-measured phylogenetic analysis using the Bayesian Markov Chain Monte Carlo (MCMC) method, as implemented in Bayesian Evolutionary Analysis Sampling Trees (BEAST) v10.5.0 [37]. Nucleotide variation was examined using the maximum likelihood-based Hasegawa-Kishino-Yano (HKY) nucleotide substitution model. The algorithm constructed a distribution of parameters estimated from the selected simulations. Data set was estimated as a tree prior with a chain length of 100 million steps with parameters sampled every 10,000 steps, and a 10% burn-in was applied. The reliability of the analysis was confirmed in Tracer v1.7.2 [37], where all parameters were found to have an effective sample size (ESS) greater than 200. Statistical uncertainty was assessed using 95% highest posterior density (HPD) values. The final tree files were visualized with FigTree v1.4.4 available from http://tree.bio.ed.ac.uk/software/figtree/.

### Discriminant analysis of principal components

The population structure of ORFV isolates, including those obtained from NCBI GenBank, was inferred using discriminant analysis of principal components (DAPC) based on the nucleotide sequences of the B2L and A32L genes. DAPC analyses were conducted using the adegenet package (ver. 2.1.11) within RStudio (R version 4.5.0). This multivariate approach is designed to delineate genetic clusters without assuming Hardy–Weinberg equilibrium or linkage equilibrium (panmixia) within groups, while simultaneously maximizing variation between groups and minimizing variation within groups. The number of principal components (PCs) retained for DAPC was determined via the a-score optimization method. The a-score was calculated ten times, and the minimum number of PCs was selected as the optimal value. Genetic clustering was subsequently evaluated using the find.clusters function of the adegenet package [38], which employs the k-means clustering algorithm and utilizes the optimal PCs as input. The maximum number of clusters (k) was determined by dividing the total number of ORFV isolates for that specific gene by 3. K-means clustering was repeated 100 times to determine the optimal number of clusters based on the lowest Bayesian information criterion (BIC), and the most frequently replicated cluster number was selected. Finally, the number of discriminant functions was set equal to the number of potential clusters minus one to produce the final clustering solution.

## Results

### Detection of orf virus in tissues by multiplex screening PCR

**Fig 2:**
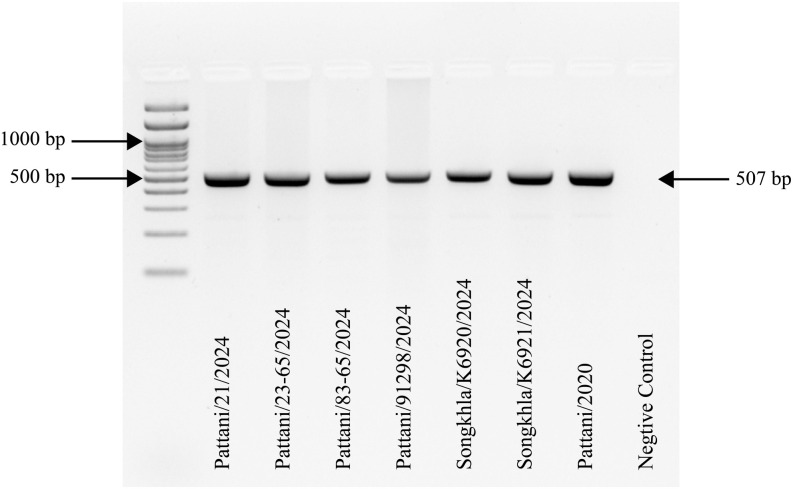
Confirmatory polymerase chain reaction (PCR) product of ORFV in all examined samples. Orf virus (ORFV) (507 base pair) was detected with gel electrophoresis using ORFV specific primer set [11]. Pattani/2020 served as positive control. Negative control was included in the last lane.

Extracted DNA from tissue samples collected as scab lesion scrapings from suspected CE animals were tested using ORFV-specific primers designed to amplify a 507 bp fragment. PCR amplification of the samples produced the expected 507 bp bands (Fig 2), confirming the presence of ORFV in all 6 suspected CE animals. Other viral infections including foot-and-mouth disease virus (FMDV), sheeppox virus, goatpox virus, bluetongue virus, and peste des petits ruminants virus were ruled out. Capripoxviruses were not detected in any of extracted DNA samples analyzed by the quantitative real-time PCR (S1 Table). DNA from the ORFV-positive samples was then amplified using gene specific primers targeting the B2L and A32L genes. Subsequently, the resulting products were sequenced and analyzed for genetic comparison.

### Genetic characterization and phylogenetic analysis

**Fig 3:**
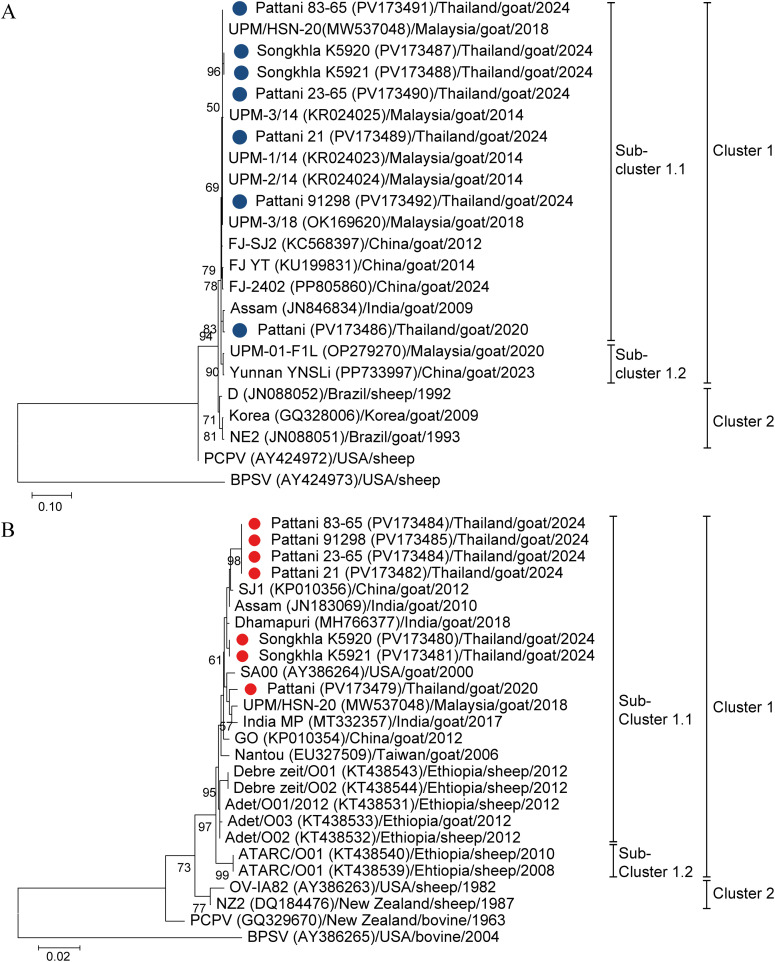
Phylogenetic analysis of orf virus isolates A) Phylogenetic tree of B2L gene. The maximum-likelihood tree of B2L gene was constructed from B2L gene nucleotide sequences. **B)** Phylogenetic analysis of A32L gene. The maximum-likelihood tree of A32L gene was constructed from A32L gene nucleotide sequences. Seven ORFV Southern Thai isolates and isolates retrieved from the GenBank database were included. The tree was generated using the T92 + G + I model with 1000 bootstrap replicates. Bootstrap values are indicated at branch nodes. Blue dots represent the ORFV B2L genes identified in this study. Red dots represent the ORFV A32L genes identified in this study. Cluster of the viruses are indicated in the right.

Genetic characterization and phylogenetic analysis based on the B2L and A32L sequences from Southern ORFV-positive isolates were conducted in comparison with previously reported sequences from other countries (Fig 3A and 3B). Genetic sequence of the positive control isolate (Pattani/Thailand/goat/2020) was also included in the analysis to provide a comprehensive genetic relatedness and evolutionary history for ORFV strains circulating across different years.

Phylogenetic analysis based on the nucleotide sequences of the B2L gene revealed that ORFV isolates from Pattani and Songkhla were grouped within the same major cluster. However, the Pattani isolate from 2020 formed a separate sub-cluster. The 2024 isolates from both Pattani and Songkhla clustered closely with Malaysian isolates from 2014 and 2018, as well as Fujian isolates from 2012, 2014, and 2024. In contrast, the Pattani 2020 isolate was grouped in a different sub-cluster alongside the Assam isolate from 2009 (Fig 3A).

We further analyzed the nucleotide sequence similarity and pairwise SNP distances of the B2L gene among Southern Thai ORFV isolates. The 2024 isolates from Pattani and Songkhla shared 99.6% nucleotide identity and 4 SNP distances. When comparing the 2024 isolates with the Pattani 2020 isolate, nucleotide similarity ranged from 98.7% to 98.9% with 11 SNP distances. The B2L gene sequences of all 2024 isolates from Pattani and Songkhla also showed high similarity (99.6%–100%) with Malaysian isolates with 2−0 SNP distances. Specifically, the 2024 Pattani isolates were most closely related to Malaysian strains including UPM-3/18 and UPM/HSN-20 of 2018, and UPM-1/14 and UPM-2/14 of 2014 (S1 and S3 Figs).

In the phylogenetic tree based on the A32L gene, the Songkhla, Pattani 2024, and Pattani 2020 isolates were grouped within the same major cluster but fell into different sub-clusters. The Songkhla and Pattani 2024 isolates clustered with the Chinese isolate from 2012, the Assam isolate from 2010, and the Dharmapuri isolate from 2018. In contrast, the Pattani 2020 isolate grouped with the Malaysian isolate from 2018, the Indian isolate from 2017, and the United States isolate from 2000 (Fig 3B).

A deletion of 4 amino acids was observed in the Songkhla isolates and the Pattani 2020 isolate, while this deletion was not present in the Pattani 2024 isolates (Fig 5). The Pattani 2020 and Songkhla 2024 isolates showed relatively low nucleotide similarity to the Pattani 2024 isolates, ranging from 84.7% to 85.3% (8–13 SNP distances), while the Songkhla isolates of 2024 shared 99.3% nucleotide identity and 5 SNP distances with the Pattani 2020 isolate (S2 and S4 Figs).

**Fig 4:**
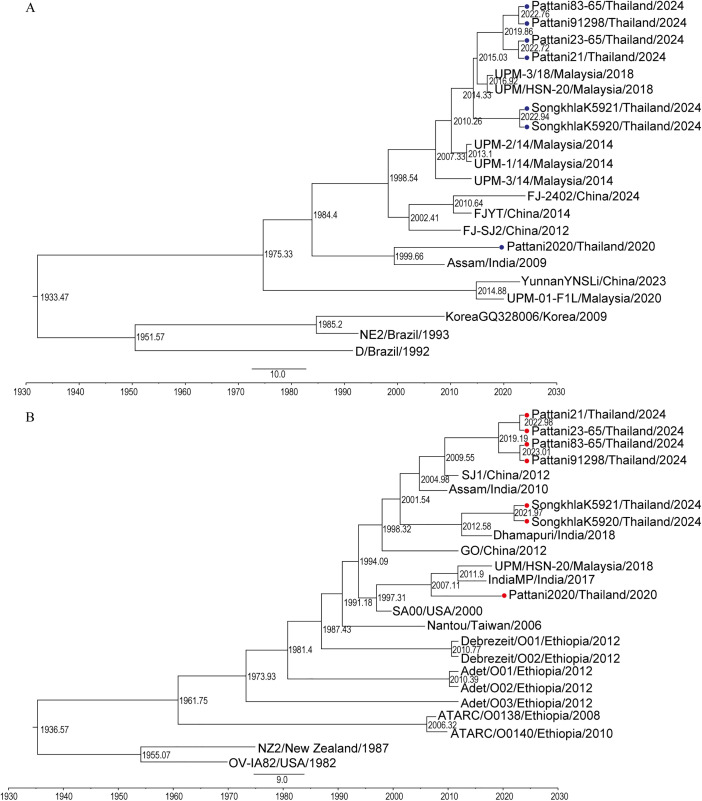
Bayesian maximum confidence (MCC) trees for the orf viruses from Southern Thailand. Bayesian phylogenetic trees were constructed for the **A)** B2L gene and **B)** A32L gene using the BEAST v1.10.5.0 software, and were visualized with FigTree (v1.4.4). The scale by factor with offset by 2024 indicates node ages on the branch nodes. The time scale is displayed at the bottom of the trees. Southern Thai isolates of the B2L gene are marked with a blue circle, while Southern Thai isolates of the A32L gene are marked with a red circle.

### Bayesian evolution analysis of orf virus isolates

To elucidate the possible circulating scenarios of the viruses in Southern region Thailand, we have analyzed a Bayesian computation of the ORFV isolates based on B2L gene and A32L gene. Based on B2L gene, Southern Thai isolates of 2024 shared common ancestry with Malaysia 2014 isolates. The Pattani isolates of 2024 shared the common ancestry with isolates of Malaysia 2018. The Songkhla isolates of 2024 also shared the common ancestry with the Malaysia 2018 isolates but cluster differently compared to the Pattani 2024 sequences. Pattani isolate of 2020 was closely related with Assam India isolate of 2009 but had different ancestry with Southern Thai isolates of 2024 based on the BEAST analysis (Fig 4A). Also, based on A32L gene, the Pattani isolate of 2020 had a distinct evolutionary path from the Southern isolates 2024. Songkhla isolates of 2024 were more closely related to the strain from India from 2018, while Pattani isolates of 2024 were more closely related to a strain from China from 2012 and India from 2010 (Fig 4B). The evolutionary rate of the B2L gene was estimated at 1.6x10^-4^ substitutions/site/year (95% HPD intervals 3.2x10^-5^, 2.9x10^-4^) and the A32L gene was estimated at 2.5x10^-4^ substitutions/site/year (95% HPD intervals 8.7x10^-5^, 4.3x10^-4^).

### Population structure of orf virus isolates

**Fig 5:**
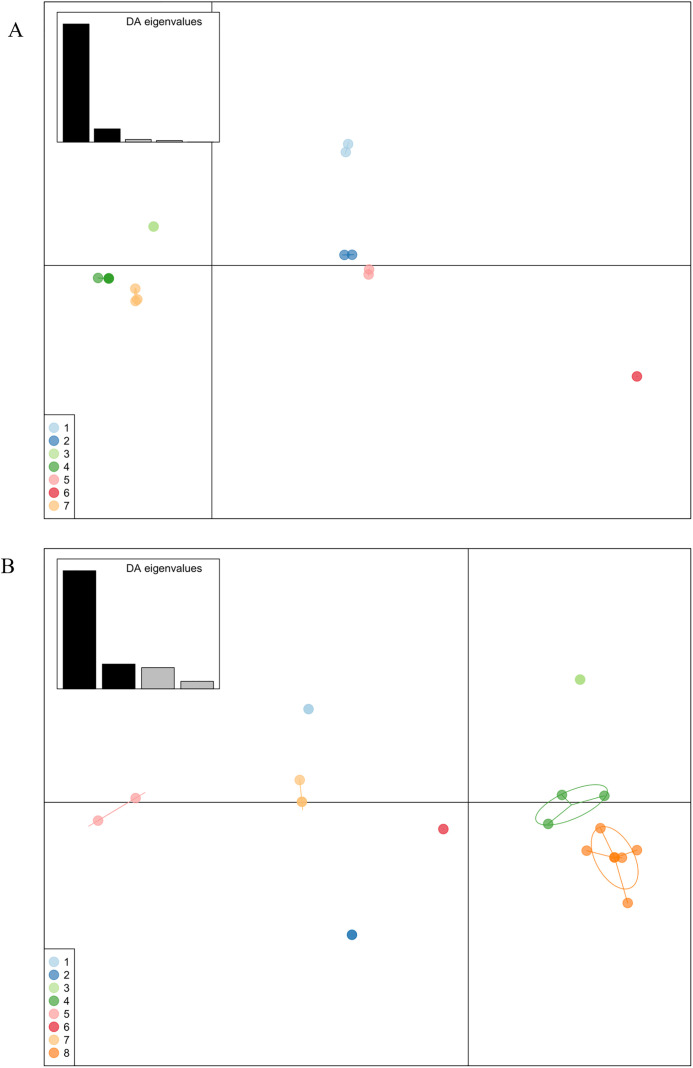
Scatter plots of ORF virus isolates based on discriminant analysis of principal components (DAPC) using A) B2L and B) A32L nucleotide sequences. Individual viral isolates are represented as dots, and groups are indicated by inertia ellipses. The bar plot of discriminant analysis (DA) eigenvalues is shown in the inset panel. The first two discriminant functions correspond to the x- and y-axes, respectively. Genetic clusters are represented in different colors. **A)** B2L gene, Cluster 1 (light blue), Korea/2009 (GQ328006) and Brazil/1993 (JN088051); Cluster 2 (dark blue), China/2023 (PP733997) and Malaysia/2020 (OP279270); Cluster 3 (light green), Songkhla/2024 (PV173487, PV173488); Cluster 4 (dark green), Pattani/2024 (PV173489–PV173492), Malaysia/2018 (OK169620, MW537048), and Malaysia/2014 (KR024023, KR024024, KR024025); Cluster 5 (light pink), Pattani/2020 (PV173486) and Assam/2009 (JN846834); Cluster 6 (dark pink), Brazil/1992 (JN088052); and Cluster 7 (light orange), China/2024 (PP805860), China/2014 (KU199831), and China/2012 (KC568397). **B)** A32L gene, Cluster 1 (light blue), Ethiopia/2012 (KT438543, KT438544); Cluster 2 (dark blue), Ethiopia/2010 (KT438540) and Ethiopia/2008 (KT438539); Cluster 3 (light green), Pattani/2024 (PV173482–PV173485); Cluster 4 (dark green), China/2012 (KP010354, KP010356) and Assam/2010 (JN183069); Cluster 5 (light pink), New Zealand/1987 (DQ184476) and USA/1982 (AY386263); Cluster 6 (dark pink), Taiwan/2006 (EU327509); Cluster 7 (light orange), Ethiopia/2012 (KT438531–KT438533); and Cluster 8 (dark orange), Songkhla/2024 (PV173480, PV173481), Pattani/2020 (PV173479), Malaysia/2018 (MW537048), India/2018 (MH766377), India/2017 (MT332357), and USA/2000 (AY386264).

The DAPC method was implemented to illustrate the population genetic clustering of ORFV isolates based on the B2L and A32L genes. For the B2L gene, a-score optimization indicated that 5 principal components (PCs), accounting for 83.5% of the conserved variance, were optimal for subsequent analysis. Seven clusters were identified after 100 replications of k-means clustering. The DAPC results revealed that the Pattani 2020 isolate (Cluster 5) was relatively distinct from the Songkhla (Cluster 3) and Pattani 2024 isolates (Cluster 4) along the first discriminant function. However, the Pattani 2020 isolate clustered with the Assam Indian 2009 isolate. All Pattani 2024 and the Malaysian isolates from 2014 and 2018 were assigned to the same cluster (Cluster 4), but separated from the Malaysia 2020 isolate (Cluster 2). A lower level of genetic differentiation was observed between the Songkhla 2024 and Pattani 2024 isolates (Fig 5A).

For the A32L gene, 4 principal components (PCs), accounting for 67.5% of the genetic variation, were retained for the DAPC analysis. The DAPC scatter plot divided the ORFV isolates into 8 distinct clusters. Cluster 3 consisted exclusively of Pattani 2024 isolates from Thailand, whereas Cluster 8 comprised ORFV isolates from multiple countries (the Pattani 2020 isolate, 2 Songkhla 2024 isolates, the Indian 2017 and 2018 isolates, the United States 2000 isolate, and the Malaysian 2018 isolate), indicating that these isolates are closely related. Cluster 8 showed a low genetic distance relative to Cluster 4, which contained the China 2012 isolates and the Assam Indian 2010 isolate (Fig 5B). Notably, the Pattani 2024 isolates in Cluster 3 were genetically indistinguishable but exhibited a slight degree of separation from Cluster 8 along the second discriminant function.

### Comparative analysis of C-terminal variability in Songkhla and Pattani isolates

The amino acid sequences were conserved in the N-terminal region of the A32L gene until the amino acid position 250. Multiple alignment of deduced amino acid sequences of the ATPase protein revealed great variation in the C-terminal region encoded by A32L gene.

**Fig 6:**
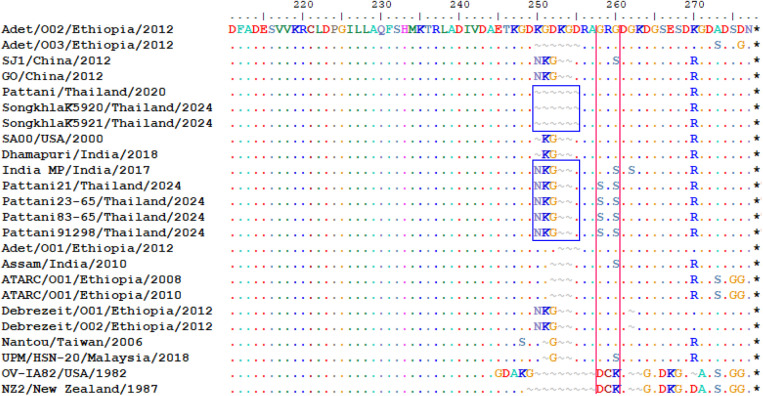
Carboxyl-terminal heterogeneity in the A32L gene-encoded ATPase of ORFV. Comparative analysis of amino acid sequences of ATPase protein of Southern Thai isolates and references isolates retrieved from Genbank were aligned using ClustalW method in Bioedit (V.7.2). The deletions of amino acid residues in Songkhla isolates from 2024 and Pattani isolate from 2020 was highlighted in blue box. The presence of unique serine (S) amino acid in Pattani isolates from 2024 were highlighted in pink box.

Twelve nucleotides deletion was observed in the Songkhla 2024 and Pattani 2020 isolates, corresponding to the deletion of 4 amino acid residues in the C-terminal region of the A32L gene. Specifically, the deletions were identified at amino acid positions 250–252 and 255 in both isolates. In contrast, the Pattani isolates of 2024 did not carry any deletions at positions 250–255 compared to Songkhla 2024 and Pattani 2020 isolates. Furthermore, the Pattani 2024 isolates carried unique amino acid substitution G258S and G260S which were not found in other Southern Thai isolates or reference sequences of the ATPase gene (Fig 6).

## Discussion

Contagious ecthyma caused by ORFV infection in goats is neglected disease in Thailand due to its low mortality rate and self-limiting nature [1,2]. In addition, its clinical signs resemble those of other viral skin diseases, making ORFV-specific diagnosis essential [10]. Previous reports of ORFV isolates were limited which led to poor understanding of the molecular epidemiology of ORFV circulation and outbreak in Thailand [22,39]. In this study, we report the first genetic sequences, molecular characterization, phylogenetic analysis and genetic structure of population of Thai ORFV isolates, specifically from the southern region based on 2 core genomic regions; the B2L and A32L genes.

The molecular analysis revealed a transboundary relationship for the recent ORFV isolates. Phylogenetic analysis based on B2L gene revealed that the Pattani and Songkhla isolates from 2024 clustered together and shared a subgroup with Malaysian isolates. This was further supported by BEAST analysis which suggested that the Southern Thai isolates of 2024 evolved from Malaysia isolates originating around 2007. To confirm population structure, we utilized the DAPC, a specialized multivariate method designed to identify and characterize clusters of genetically related entities by maximizing differentiation between groups while minimizing variation within groups [40]. The clusters identified by the DAPC analysis were consistent with the phylogenetic results, grouping the Pattani 2024 isolates and Malaysian isolates from 2014 and 2018. Given that previous studies have reported ORFV outbreaks in neighboring Malaysia [12,17,18], a country bordering Southern Thailand, it’s possible for the viruses to spread between these neighboring countries.

Although Pattani and Songkhla isolates from 2024 showed high overall genetic relatedness, the phylogenetic and DAPC analysis revealed local differentiation, indicating they are different viruses. The phylogenetic and DPAC analysis identified different clusters for the 2024 isolates based on B2L gene, indicating a low level of genetic differentiation in circulation. This different clustering is in line with the BEAST analysis suggesting a slight genetic evolution of the circulating viruses. Similarly, the phylogenetic analysis of the A32L gene showed that the Pattani and Songkhla isolates from 2024 also clustered together but within different sub branch. The DAPC analysis of A32L further revealed a distinct cluster for the Pattani 2024 isolates, which may reflect the unique amino acid substitutions.

Significantly, the Pattani 2020 isolate presented a separate evolutionary profile. Based on phylogenetic analysis, this isolate fell into a different subgroup for both B2L and A32L genes, despite belonging to the same main cluster. Moreover, BEAST analysis indicated that the Pattani 2020 isolates has a different ancestral origin compared with the recent circulating Southern Thai isolates, suggesting a distinct introduction event of ORFV into the region. Together, the genetic and evolutionary analysis of the ORFV isolates suggests the sustained circulation and evolution of the ORFV in Southern Thailand, with mutation accumulating in the regional goat population prior to their identification.

The ORFV isolates from Southern Thailand exhibit genetic variation. Despite the high B2L gene similarity between the Songkhla and Pattani 2024 isolates, their A32L gene sequences showed lower similarity. This difference in nucleotide similarity is due to a 12-nucleotide deletion corresponding to a 4-amino-acid deletion detected in the Songkhla 2024 and Pattani 2020 isolates. These deletions located between amino acid positions 250–255 have also been observed in the Adet/O03/2012 strain from Ethiopia [16]. Although studies have shown nucleotide deletions of ORFV sequences in ATPase protein residing within C-terminal regions [16,28,41], the impact of the observed deletions and substitutions remains unclear. A32L gene encodes for ATPase that hydrolyzes ATP to provide energy for the packaging of viral DNA [31,32,42]. Thus, it could be speculated that the deletions may alter the protein’s enzymatic activity or its interactions with other proteins affecting affect viral DNA packaging [28]. In addition, Pattani isolates of 2024 carry unique amino acid substitutions (G258S and G260S) at the C-terminal regions of ATPase protein. Whether these residues substitution could contribute to genetic variation from other Southern isolates and global isolates remain unknown. However, some studies suggested the unique amino acid substitutions might associated with host specificity, pathogenicity or virulence, yet these needed to be confirmed by additional in vitro and in vivo studies [41,43].

Collectively, the genetic and evolutionary evidence of the ORFV isolates from Southern Thailand demonstrate sustained circulation and evolution with mutations accumulating in the regional goat population before their identification. This study, despites its limited small sample size, revealed consistent findings across different analytical principles. The sequences closely resemble international strains in the B2L and A32L genes but carry unique deletions and substitutions indicating genetic variation of the viruses. The identified amino acid substitutions and deletions in the C-terminal region of the A32L gene may serve as a target for future functional studies. Continuous surveillance coupled with complete genome sequencing and multi-gene analysis will provide a better understanding of the genetic diversity of ORFV circulating in Thailand. This larger genomic dataset will be valuable for network analyses to investigate ORFV viral transmission dynamics and connections among circulating strains within the region.

## Supporting information

S1 Fig Nucleotide identities of orf virus nucleotide sequences based on B2L gene. The nucleotide identity was generated using Bioedit software (V.7.2).
(PDF)

S2 Fig Nucleotide identities of orf virus nucleotide sequences based on A32L gene. The nucleotide identity was generated using Bioedit software (V.7.2).
(PDF)

S3 Fig Pairwise SNP differences of orf viruses based on B2L gene. The SNP differences were calculated with snp-dists (v0.8.2). The numbers in the table show number of SNP distance.
(PDF)

S4 Fig Pairwise SNP differences of orf viruses based on A32L gene. The SNP differences were calculated with snp-dists (v0.8.2). The numbers in the table show number of SNP distance.
(PDF)

S1 Table Detection of capripoxvirus by quantitative real-time PCR (qPCR) using DNA extracted from seven scabby goat tissues. The absolute quantification of capripoxvirus using capripox gene-specific primers and probe was determined by the quantitative real-time PCR (qPCR soft, Analytik jena). DNA sample from Lumpy skin disease virus isolate 144240/64 was used as a positive control.
(PDF)

S1_raw_images Original gel image. Uncropped gel from Fig 2. Electrophoresis of the positive PCR products showed a band of 507 base pairs. Lanes not included in the final figure were marked as X.
(TIF)
