# Supplementary material for: Molecular characterization and phylogenetic analysis of major envelope protein gene (B2L) and ATPase protein gene (A32L) of orf virus isolates from goats in Southern, Thailand
Source: PLoS One. 2026 Jan 30;21(1):e0340195. doi: 10.1371/journal.pone.0340195 (PMC12857932; doi:10.1371/journal.pone.0340195)
Supplement: S1 Fig — The nucleotide identity was generated using Bioedit software (V.7.2). (PDF) [file pone.0340195.s001.pdf]

Seq->	Korea (GQ328006)/Korea/goat/2009	NE2 (JN088051)/Brazil/goat/1993	D (JN088052)/Brazil/sheep/1992	Assam(JN846834)/India/goat/2009	UPM-3/18 (OK169620)/Malaysia/goat/2018	FJ-SJ2 (KC568397)/China/goat/2012	FJ YT(KU199831)/China/goat/2014	UPM-01-F1L (OP279270)/Malaysia/goat/2020	Yunnan /YNSLi (PP733997)/China/goat/2023	FJ-2402(PP805860)/China/goat/2024	Pattani 23-65 (PV173490)/Thailand/goat/2024	Pattani 83-65 (PV173491)/Thailand/goat/2024	Pattani 21 (PV173489)/Thailand/goat/2024	Pattani 91298 (PV173492)/Thailand/goat/2024	Pattani(PV173486)/Thailand/goat/2020	Songkhla K5920(PV173487)/Thailand/goat/2024	Songkhla K5921(PV173488)/Thailand/goat/2024	UPM-1/14 (KR024023)/Malaysia/goat/2014	UPM-3/14 (KR024025)/Malaysia/goat/2014	UPM-2/14 (KR024024)/Malaysia/goat/2014	UPM/HSN-20(MW537048)/Malaysia/goat/2018	BPSV (AY424973)/USA/sheep	PCPV (AY424972)/USA/sheep
Korea (GQ328006)/Korea/goat/2009	ID	99.5	98.7	97.5	97.9	97.8	97.7	98.3	98.3	97.7	97.9	97.9	97.9	97.9	97.6	97.9	97.9	97.9	97.7	97.9	97.9	84.1	94.9
NE2 (JN088051)/Brazil/goat/1993	99.5	ID	98.6	97.8	98.2	98.1	98.0	98.8	98.8	98.0	98.2	98.2	98.2	98.2	98.1	98.2	98.2	98.2	98.0	98.2	98.2	84.1	95.0
D (JN088052)/Brazil/sheep/1992	98.7	98.6	ID	98.0	98.2	98.1	98.0	98.0	98.0	98.0	98.2	98.2	98.2	98.2	97.7	98.0	98.0	98.2	98.0	98.2	98.2	84.1	95.4
Assam(JN846834)/India/goat/2009	97.5	97.8	98.0	ID	99.0	98.7	98.8	98.4	98.4	98.8	99.0	99.0	99.0	99.0	99.3	98.8	98.8	99.0	98.8	99.0	99.0	83.9	95.0
UPM-3/18 (OK169620)/Malaysia/goat/2018	97.9	98.2	98.2	99.0	ID	99.7	99.6	99.0	99.0	99.6	100.0	100.0	100.0	100.0	98.9	99.6	99.6	100.0	99.8	100.0	100.0	84.1	95.4
FJ-SJ2 (KC568397)/China/goat/2012	97.8	98.1	98.1	98.7	99.7	ID	99.5	98.9	98.9	99.5	99.7	99.7	99.7	99.7	98.8	99.3	99.3	99.7	99.5	99.7	99.7	84.1	95.3
FJ YT(KU199831)/China/goat/2014	97.7	98.0	98.0	98.8	99.6	99.5	ID	98.8	98.8	99.8	99.6	99.6	99.6	99.6	98.9	99.2	99.2	99.6	99.4	99.6	99.6	84.0	95.2
UPM-01-F1L (OP279270)/Malaysia/goat/2020	98.3	98.8	98.0	98.4	99.0	98.9	98.8	ID	99.8	98.8	99.0	99.0	99.0	99.0	99.1	99.0	99.0	99.0	98.8	99.0	99.0	84.3	95.0
Yunnan /YNSLi (PP733997)/China/goat/2023	98.3	98.8	98.0	98.4	99.0	98.9	98.8	99.8	ID	98.8	99.0	99.0	99.0	99.0	98.9	99.0	99.0	99.0	98.8	99.0	99.0	84.5	95.2
FJ-2402(PP805860)/China/goat/2024	97.7	98.0	98.0	98.8	99.6	99.5	99.8	98.8	98.8	ID	99.6	99.6	99.6	99.6	98.9	99.4	99.4	99.6	99.4	99.6	99.6	83.9	95.2
Pattani 23-65 (PV173490)/Thailand/goat/2024	97.9	98.2	98.2	99.0	100.0	99.7	99.6	99.0	99.0	99.6	ID	100.0	100.0	100.0	98.9	99.6	99.6	100.0	99.8	100.0	100.0	84.1	95.4
Pattani 83-65 (PV173491)/Thailand/goat/2024	97.9	98.2	98.2	99.0	100.0	99.7	99.6	99.0	99.0	99.6	100.0	ID	100.0	100.0	98.9	99.6	99.6	100.0	99.8	100.0	100.0	84.1	95.4
Pattani 21 (PV173489)/Thailand/goat/2024	97.9	98.2	98.2	99.0	100.0	99.7	99.6	99.0	99.0	99.6	100.0	100.0	ID	100.0	98.9	99.6	99.6	100.0	99.8	100.0	100.0	84.1	95.4
Pattani 91298 (PV173492)/Thailand/goat/2024	97.9	98.2	98.2	99.0	100.0	99.7	99.6	99.0	99.0	99.6	100.0	100.0	100.0	ID	98.9	99.6	99.6	100.0	99.8	100.0	100.0	84.1	95.4
Pattani(PV173486)/Thailand/goat/2020	97.6	98.1	97.7	99.3	98.9	98.8	98.9	99.1	98.9	98.9	98.9	98.9	98.9	98.9	ID	98.7	98.7	98.9	98.7	98.9	98.9	84.1	95.2
Songkhla K5920(PV173487)/Thailand/goat/2024	97.9	98.2	98.0	98.8	99.6	99.3	99.2	99.0	99.0	99.4	99.6	99.6	99.6	99.6	98.7	ID	100.0	99.6	99.4	99.6	99.6	84.2	95.1
Songkhla K5921(PV173488)/Thailand/goat/2024	97.9	98.2	98.0	98.8	99.6	99.3	99.2	99.0	99.0	99.4	99.6	99.6	99.6	99.6	98.7	100.0	ID	99.6	99.4	99.6	99.6	84.2	95.1
UPM-1/14 (KR024023)/Malaysia/goat/2014	97.9	98.2	98.2	99.0	100.0	99.7	99.6	99.0	99.0	99.6	100.0	100.0	100.0	100.0	98.9	99.6	99.6	ID	99.8	100.0	100.0	84.1	95.4
UPM-3/14 (KR024025)/Malaysia/goat/2014	97.7	98.0	98.0	98.8	99.8	99.5	99.4	98.8	98.8	99.4	99.8	99.8	99.8	99.8	98.7	99.4	99.4	99.8	ID	99.8	99.8	84.0	95.2
UPM-2/14 (KR024024)/Malaysia/goat/2014	97.9	98.2	98.2	99.0	100.0	99.7	99.6	99.0	99.0	99.6	100.0	100.0	100.0	100.0	98.9	99.6	99.6	100.0	99.8	ID	100.0	84.1	95.4
UPM/HSN-20(MW537048)/Malaysia/goat/2018	97.9	98.2	98.2	99.0	100.0	99.7	99.6	99.0	99.0	99.6	100.0	100.0	100.0	100.0	98.9	99.6	99.6	100.0	99.8	100.0	ID	84.1	95.4
BPSV (AY424973)/USA/sheep	84.1	84.1	84.1	83.9	84.1	84.1	84.0	84.3	84.5	83.9	84.1	84.1	84.1	84.1	84.1	84.2	84.2	84.1	84.0	84.1	84.1	ID	85.0
PCPV (AY424972)/USA/sheep	94.9	95.0	95.4	95.0	95.4	95.3	95.2	95.0	95.2	95.2	95.4	95.4	95.4	95.4	95.2	95.1	95.1	95.4	95.2	95.4	95.4	85.0	ID
