# Supplementary material for: Molecular characterization and phylogenetic analysis of major envelope protein gene (B2L) and ATPase protein gene (A32L) of orf virus isolates from goats in Southern, Thailand
Source: PLoS One. 2026 Jan 30;21(1):e0340195. doi: 10.1371/journal.pone.0340195 (PMC12857932; doi:10.1371/journal.pone.0340195)
Supplement: S3 Fig — The SNP differences were calculated with snp-dists (v0.8.2). The numbers in the table show number of SNP distance. (PDF) [file pone.0340195.s003.pdf]

SNP distance	FJ YT(KU199831)/China/goat/2014	FJ-2402(PP805860)/China/goat/2024	Assam(JN846834)/India/goat/2009	Korea (GQ328006)/Korea/goat/2009	**Pattani(PV173486)/Thailand/goat/2020**	UPM-01-F1L (OP279270)/Malaysia/goat/2020	Yunnan YNSLi (PP733997)/China/goat/2023	D (JN088052)/Brazil/sheep/1992	NE2 (JN088051)/Brazil/goat/1993	FJ-SJ2 (KC568397)/China/goat/2012	**Songkhla K5920(PV173487)/Thailand/goat/2024**	**Songkhla K5921(PV173488)/Thailand/goat/2024**	UPM-3/14 (KR024025)/Malaysia/goat/2014	UPM-3/18 (OK169620)/Malaysia/goat/2018	**Pattani 23-65 (PV173490)/Thailand/goat/2024**	**Pattani 83-65 (PV173491)/Thailand/goat/2024**	**Pattani 21 (PV173489)/Thailand/goat/2024**	**Pattani 91298 (PV173492)/Thailand/goat/2024**	UPM-1/14 (KR024023)/Malaysia/goat/2014	UPM-2/14 (KR024024)/Malaysia/goat/2014	UPM/HSN-20(MW537048)/Malaysia/goat/2018
FJ YT(KU199831)/China/goat/2014	0	2	12	23	11	12	12	20	20	5	8	8	6	4	4	4	4	4	4	4	4
FJ-2402(PP805860)/China/goat/2024	2	0	12	23	11	12	12	20	20	5	6	6	6	4	4	4	4	4	4	4	4
Assam(JN846834)/India/goat/2009	12	12	0	25	7	16	16	20	22	13	12	12	12	10	10	10	10	10	10	10	10
Korea (GQ328006)/Korea/goat/2009	23	23	25	0	24	17	17	13	5	22	21	21	23	21	21	21	21	21	21	21	21
**Pattani(PV173486)/Thailand/goat/2020**	11	11	7	24	0	9	11	23	19	12	13	13	13	11	11	11	11	11	11	11	11
UPM-01-F1L (OP279270)/Malaysia/goat/2020	12	12	16	17	9	0	2	20	12	11	10	10	12	10	10	10	10	10	10	10	10
Yunnan YNSLi (PP733997)/China/goat/2023	12	12	16	17	11	2	0	20	12	11	10	10	12	10	10	10	10	10	10	10	10
D (JN088052)/Brazil/sheep/1992	20	20	20	13	23	20	20	0	14	19	20	20	20	18	18	18	18	18	18	18	18
NE2 (JN088051)/Brazil/goat/1993	20	20	22	5	19	12	12	14	0	19	18	18	20	18	18	18	18	18	18	18	18
FJ-SJ2 (KC568397)/China/goat/2012	5	5	13	22	12	11	11	19	19	0	7	7	5	3	3	3	3	3	3	3	3
**Songkhla K5920(PV173487)/Thailand/goat/2024**	8	6	12	21	13	10	10	20	18	7	0	0	6	4	4	4	4	4	4	4	4
**Songkhla K5921(PV173488)/Thailand/goat/2024**	8	6	12	21	13	10	10	20	18	7	0	0	6	4	4	4	4	4	4	4	4
UPM-3/14 (KR024025)/Malaysia/goat/2014	6	6	12	23	13	12	12	20	20	5	6	6	0	2	2	2	2	2	2	2	2
UPM-3/18 (OK169620)/Malaysia/goat/2018	4	4	10	21	11	10	10	18	18	3	4	4	2	0	0	0	0	0	0	0	0
**Pattani 23-65 (PV173490)/Thailand/goat/2024**	4	4	10	21	11	10	10	18	18	3	4	4	2	0	0	0	0	0	0	0	0
**Pattani 83-65 (PV173491)/Thailand/goat/2024**	4	4	10	21	11	10	10	18	18	3	4	4	2	0	0	0	0	0	0	0	0
**Pattani 21 (PV173489)/Thailand/goat/2024**	4	4	10	21	11	10	10	18	18	3	4	4	2	0	0	0	0	0	0	0	0
**Pattani 91298 (PV173492)/Thailand/goat/2024**	4	4	10	21	11	10	10	18	18	3	4	4	2	0	0	0	0	0	0	0	0
UPM-1/14 (KR024023)/Malaysia/goat/2014	4	4	10	21	11	10	10	18	18	3	4	4	2	0	0	0	0	0	0	0	0
UPM-2/14 (KR024024)/Malaysia/goat/2014	4	4	10	21	11	10	10	18	18	3	4	4	2	0	0	0	0	0	0	0	0
UPM/HSN-20(MW537048)/Malaysia/goat/2018	4	4	10	21	11	10	10	18	18	3	4	4	2	0	0	0	0	0	0	0	0

No. of SNP distance
0
2
3
4
5
6
7
8
9
10
11
12
13
14
16
17
18
19
20
21
22
23
24
25
