# Supplementary material for: Molecular characterization and phylogenetic analysis of major envelope protein gene (B2L) and ATPase protein gene (A32L) of orf virus isolates from goats in Southern, Thailand
Source: PLoS One. 2026 Jan 30;21(1):e0340195. doi: 10.1371/journal.pone.0340195 (PMC12857932; doi:10.1371/journal.pone.0340195)
Supplement: S4 Fig — The SNP differences were calculated with snp-dists (v0.8.2). The numbers in the table show number of SNP distance. (PDF) [file pone.0340195.s004.pdf]

snp-dists 0.8.2	Adet/O02/2012(KT438532)/Ethiopia/sheep/2012	Adet/O01/2012(KT438531)/Ethiopia/sheep/2012	Adet/O03/2012(KT438533)/Ethiopia/goat/2012	ATARC/O01(KT438539)/Ethiopia/sheep/2008	ATARC/O01(KT438540)/Ethiopia/sheep/2010	OV-IA82(AY386263)/USA/sheep/1982	NZ2(DQ184476)/New_Zealand/sheep/1987	Nantou(EU327509)/Taiwan/goat/2006	Assam(JN183069)/India/goat/2010	**Pattani23-65(PV173483)/Thailand/goat/2024**	**Pattani 83-65 (PV173484)/Thailand/goat/2024**	**Pattani21(PV173482)/Thailand/goat/2024**	**Pattani91298(PV173485)/Thailand/goat/2024**	SJ1(KP010356)/China/goat/2012	Dhamapuri(MH766377)/India/goat/2018	**SongkhlaK5920(PV173480)/Thailand/goat/2024**	**SongkhlaK5921(PV173481)/Thailand/goat/2024**	GO(KP010354)/China/goat/2012	Debrezeit/O01(KT438543)/Ethiopia/sheep/2012	Debrezeit/O02(KT438544)/Ethiopia/sheep/2012	SA00(AY386264)/USA/goat/2000	**Pattani(PV173479)/Thailand/goat/2020**	IndiaMP(MT332357)/India/goat/2017	UPM/HSN-20(MW537048)/Malaysia/goat/2018
Adet/O02/2012(KT438532)/Ethiopia/sheep/2012	0	0	3	12	12	31	19	7	6	13	13	13	13	11	11	6	6	11	8	8	11	9	14	10
Adet/O01/2012(KT438531)/Ethiopia/sheep/2012	0	0	3	12	12	31	19	7	6	13	13	13	13	11	11	6	6	11	8	8	11	9	14	10
Adet/O03/2012(KT438533)/Ethiopia/goat/2012	3	3	0	11	11	30	18	9	9	11	11	11	11	9	10	9	9	9	6	6	10	12	12	12
ATARC/O01(KT438539)/Ethiopia/sheep/2008	12	12	11	0	0	32	21	16	14	20	20	20	20	18	18	14	14	18	19	19	18	16	20	16
ATARC/O01(KT438540)/Ethiopia/sheep/2010	12	12	11	0	0	32	21	16	14	20	20	20	20	18	18	14	14	18	19	19	18	16	20	16
OV-IA82(AY386263)/USA/sheep/1982	31	31	30	32	32	0	13	34	33	35	35	35	35	34	36	36	36	35	32	32	37	39	36	36
NZ2(DQ184476)/New_Zealand/sheep/1987	19	19	18	21	21	13	0	25	22	24	24	24	24	23	26	25	25	24	22	22	26	28	25	25
Nantou(EU327509)/Taiwan/goat/2006	7	7	9	16	16	34	25	0	8	13	13	13	13	9	9	8	8	9	10	10	9	11	12	11
Assam(JN183069)/India/goat/2010	6	6	9	14	14	33	22	8	0	8	8	8	8	6	8	4	4	8	12	12	10	9	11	5
**Pattani23-65(PV173483)/Thailand/goat/2024**	13	13	11	20	20	35	24	13	8	0	0	0	0	6	9	8	8	8	10	10	11	13	11	10
**Pattani 83-65 (PV173484)/Thailand/goat/2024**	13	13	11	20	20	35	24	13	8	0	0	0	0	6	9	8	8	8	10	10	11	13	11	10
**Pattani21(PV173482)/Thailand/goat/2024**	13	13	11	20	20	35	24	13	8	0	0	0	0	6	9	8	8	8	10	10	11	13	11	10
**Pattani91298(PV173485)/Thailand/goat/2024**	13	13	11	20	20	35	24	13	8	0	0	0	0	6	9	8	8	8	10	10	11	13	11	10
SJ1(KP010356)/China/goat/2012	11	11	9	18	18	34	23	9	6	6	6	6	6	0	5	4	4	6	9	9	7	7	7	8
Dhamapuri(MH766377)/India/goat/2018	11	11	10	18	18	36	26	9	8	9	9	9	9	5	0	3	3	7	10	10	8	8	10	10
**SongkhlaK5920(PV173480)/Thailand/goat/2024**	6	6	9	14	14	36	25	8	4	8	8	8	8	4	3	0	0	6	9	9	5	5	7	7
**SongkhlaK5921(PV173481)/Thailand/goat/2024**	6	6	9	14	14	36	25	8	4	8	8	8	8	4	3	0	0	6	9	9	5	5	7	7
GO(KP010354)/China/goat/2012	11	11	9	18	18	35	24	9	8	8	8	8	8	6	7	6	6	0	8	8	7	9	9	6
Debrezeit/O01(KT438543)/Ethiopia/sheep/2012	8	8	6	19	19	32	22	10	12	10	10	10	10	9	10	9	9	8	0	0	10	12	11	12
Debrezeit/O02(KT438544)/Ethiopia/sheep/2012	8	8	6	19	19	32	22	10	12	10	10	10	10	9	10	9	9	8	0	0	10	12	11	12
SA00(AY386264)/USA/goat/2000	11	11	10	18	18	37	26	9	10	11	11	11	11	7	8	5	5	7	10	10	0	8	8	8
**Pattani(PV173479)/Thailand/goat/2020**	9	9	12	16	16	39	28	11	9	13	13	13	13	7	8	5	5	9	12	12	8	0	8	8
IndiaMP(MT332357)/India/goat/2017	14	14	12	20	20	36	25	12	11	11	11	11	11	7	10	7	7	9	11	11	8	8	0	7
UPM/HSN-20(MW537048)/Malaysia/goat/2018	10	10	12	16	16	36	25	11	5	10	10	10	10	8	10	7	7	6	12	12	8	8	7	0

**No. of SNP distance**
0
3
4
5
6
7
8
9
10
11
12
13
14
16
18
19
20
21
22
23
24
25
26
28
30
31
32
33
34
35
36
37
39
