# Supplementary material for: Molecular characterization and phylogenetic analysis of major envelope protein gene (B2L) and ATPase protein gene (A32L) of orf virus isolates from goats in Southern, Thailand
Source: PLoS One. 2026 Jan 30;21(1):e0340195. doi: 10.1371/journal.pone.0340195 (PMC12857932; doi:10.1371/journal.pone.0340195)
Supplement: S1 Table — The absolute quantification of capripoxvirus using capripox gene-specific primers and probe was determined by the quantitative real-time PCR (qPCR soft, Analytik jena). DNA sample from Lumpy skin disease virus isolate 144240/64 was used as a positive control. (PDF) [file pone.0340195.s005.pdf]

**Isolate**	**Ct value**	**Result**
Pattani 2020/Thailand/goat/2020	Undetermined	Negative
Songkhla K5920/ Thailand/goat/2024	Undetermined	Negative
Songkhla K5921/Thailand/goat/2024	Undetermined	Negative
Pattani 21/Thailand/goat/2024	Undetermined	Negative
Pattani 23-65/Thailand/goat/2024	Undetermined	Negative
Pattani 83-65/Thailand/goat/2024	Undetermined	Negative
Pattani 91298 /Thailand/goat/2024	Undetermined	Negative
Positive control (Lumpy skin disease virus isolate 144240/64)	32.07	Positive
Negative control (Distilled water)	Undetermined	Negative
