# Supplementary figures and images for: Molecular characterization and phylogenetic analysis of major envelope protein gene (B2L) and ATPase protein gene (A32L) of orf virus isolates from goats in Southern, Thailand
Source: PLoS One. 2026 Jan 30;21(1):e0340195. doi: 10.1371/journal.pone.0340195 (PMC12857932; doi:10.1371/journal.pone.0340195)

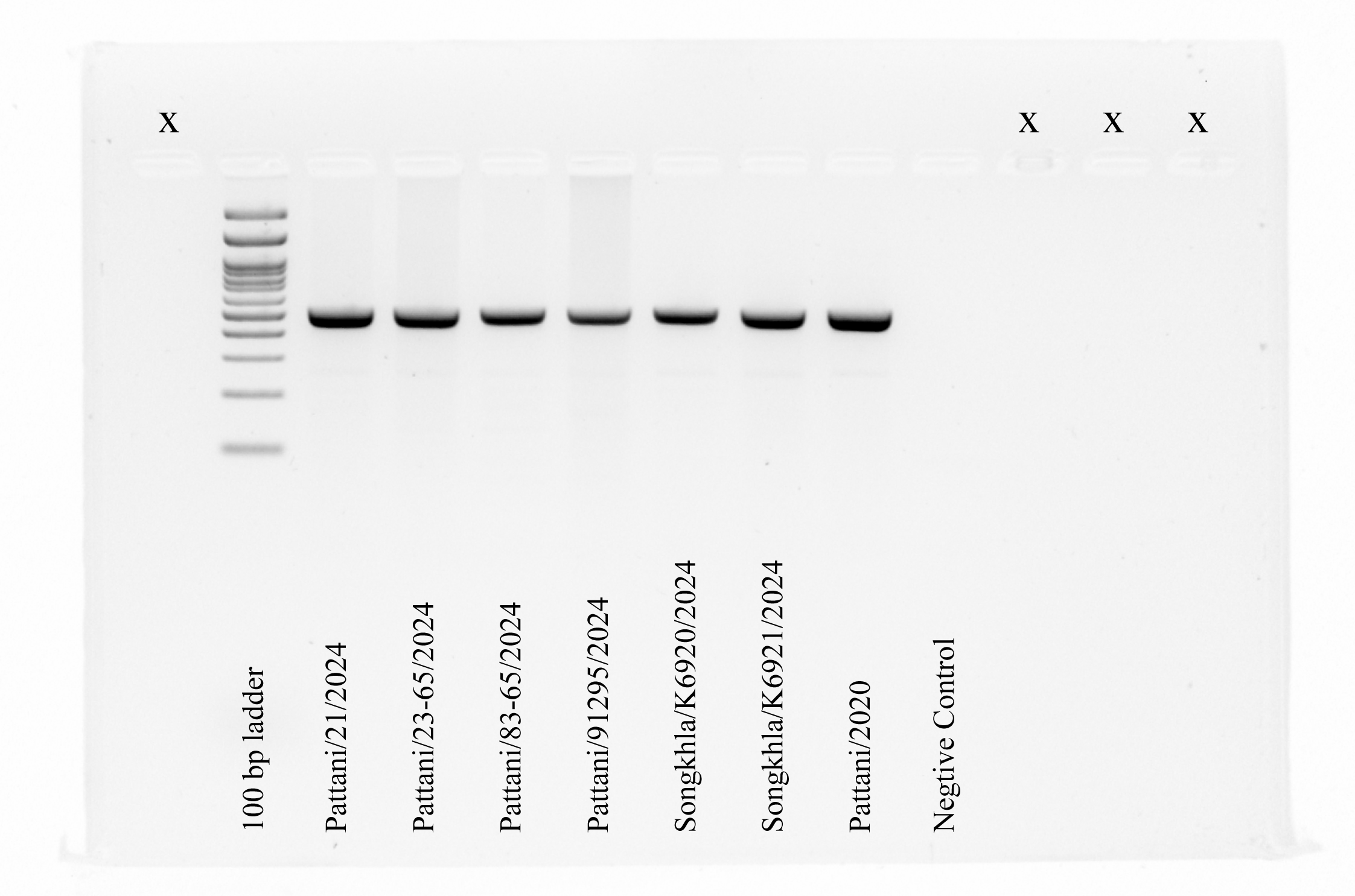

Supplement: S1_raw_images — Uncropped gel from Fig 2. Electrophoresis of the positive PCR products showed a band of 507 base pairs. Lanes not included in the final figure were marked as X. (TIF) [file pone.0340195.s006.tif]
